# Use of deferred consent for severely ill children in a multi-centre phase III trial

**DOI:** 10.1186/1745-6215-12-90

**Published:** 2011-03-31

**Authors:** Kathryn Maitland, Sassy Molyneux, Mwamvua Boga, Sarah Kiguli, Trudie Lang

**Affiliations:** 1 Kemri-Wellcome Centre for Geographic medicine, coast, Kenya; 2 Department of Paediatrics and Child Health Makerere University. Uganda; 3 Centre for Tropical Medicine, University of Oxford

## Abstract

**Background:**

Voluntary participation of a subject in research respects a subject's rights, strengthens its ethical conduct, and is formalized by the informed consent process. Clinical trials of life-saving interventions for medical emergencies often necessitate enrolment of patients where prior written individual informed consent is impossible. Although there are regulations and guidelines on protecting subjects in emergency research, these have been criticised for being limited and unnecessarily restrictive. Across Europe and the United States stringent regulations have resulted in a substantial decline of clinical trials involving emergency interventions.

**Methods:**

We are conducting a trial of fluid resuscitation in children with hypovolaemic shock in six hospitals across three malaria-endemic African countries. The design is pragmatic as children are enrolled on clinical criteria alone and is being conducted in hospitals with facilities typical of many district hospitals across Africa. The trial aims to inform strategy for managing children with febrile illness and features of shock. In order to develop appropriate consent processes for the trial, we conducted a narrative review of current international recommendations for emergency consent.

**Results:**

Practical or specific guidance was generally sparse or confusing with few examples in the literature to direct our informed consent process. For a sub-group of children who were critically sick or where parents themselves were otherwise too distressed to consider prior written consent, we opted for a modified form of deferred consent. This included verbal assent from guardians at the point of enrolment, with full written consent obtained after stabilising the child. For children who died prior to full written consent, ethical permission was received to waiver full consent.

**Conclusions:**

In light of the controversy around guidance and regulations in this area we report how and why we have used a modified system of deferred consent in an emergency intervention trial in children. Although approved by all relevant ethics committees and operational in 3 countries in Africa, formal research is now necessary to explore the perceptions and experiences of parents, health workers, researchers and ethics committees of the modified method of deferred consent.

## Background

Respect for persons, beneficence, and justice are the principles underlying guidelines on the ethical conduct of research in human subjects. The voluntary participation of a subject in a research study respects a subject's rights, and is formalized by the informed consent process. In therapeutic studies and clinical trials in severely ill children and emergency care the challenges for researchers regarding fully informed consent are not trivial. In these situations the requirements for the protection of human subjects with diminished autonomy presents unique and complex challenges. Although seemly addressed by current international guidelines and regulations, there are concerns that the recommendations on the exceptions to informed consent, and the interpretation of these by ethics committees, are too restrictive [1-3]. As a consequence obtaining ethics approval for resuscitation and emergency therapeutic intervention trials is challenging, and trials of emergency interventions are becoming increasingly uncommon[4,5]. The major difficulty is how to approach informed consent in research involving severely sick children in a way that is appropriate, practical and ethical whilst acceptable to regulatory bodies and ethics committees.

## Methods

Fluid Expansion As Supportive Therapy (FEAST) in critically ill African children trial is underway in six hospitals across three African countries [6]. The trial examines three different fluid resuscitation strategies on 48 hour and 28 day survival. The design is pragmatic as children are enrolled on clinical criteria alone and it is being conducted in hospitals with facilities typical of many district hospitals across sub Saharan Africa. The main objective of the trial is to facilitate clinicians and policy makers working in sub-Saharan Africa in deciding the best strategy to adopt in managing children with febrile illness and features of shock.

For this late phase clinical trial of fluid resuscitation http://feast-trial.org/ or ISCTRN 69856593 we conducted a narrative review of international recommendations and guidelines, and of empirical literature, to inform our process of consent. Following a detailed review of international regulatory body requirements in these settings, we searched the academic literature through combining keywords in pubmed.org including "emergency", "consent", "clinical trial", "assent", "waiver", "paediatric", "community consultation "and "children". In particular, we considered two important practical and ethical aspects of the consent process: community consultation, and alternatives to prior full written consent among populations with diminished autonomy. Information from this review fed into consent discussions in trial planning meetings.

## Results

### The Current International Regulatory Situation

The regulatory environment differs slightly between Europe and the United States of America (US). The European clinical trial directive states that consent must be given on behalf of a minor *prior to research commencing*[7]. When this directive became law across Europe it effectively resulted in a halt to emergency research in children [8]. A few European countries have since amended this law at national level to allow for emergency research [9,10]. In the US, there was no provision in their regulations that allowed any exception from the informed consent requirement, for greater than minimal risk emergency research. New regulations, brought in 1996, by the United States Food and Drug Administration (FDA)provided an opportunity for research to proceed without consent and designated such exemptions from informed consent (EFIC) as "the Final Rule''[11]. More than a decade later little progress has been made in the study of therapies for acute, life-threatening conditions with high mortality rates[12].

The key elements of the EFIC Final Rule include[13]: 1) the subject has an immediately life-threatening condition; 2) available treatments are unproven or unsatisfactory; 3) consent from the subject (or a surrogate) is not feasible due to the urgency of the patient/subject's condition; 4) the research could not otherwise be performed; (5) the risks and benefits are reasonable; and (6) there is a prospect that the trial will be of direct medical benefit to the patient/subject. An additional requirement is consultation with the community from which subjects will be drawn, including public disclosure of the study design and risks prior to commencement. As with all research, it is also recommended that individual clinical results are fed back continuously, and that research findings are fed back when the study is over. Studies have shown that the goals of the current regulatory requirement, although well-conceived, were not being met [14]. One example, highlighted was the ineffectiveness of community consultation efforts.

Guidelines on emergency consent elsewhere in the world vary or are not specifically addressed. For example, South African Medical Research Council's guidelines make no specific recommendations for research in emergency situations[15]. CIOMS Guidelines on Biomedical Research[3] and the UNESCO Declaration on Biomedicine[16] make clear that any waiver or delayed consent should be a rare exception and subject to full ethics committee review and must bring direct benefit to the participant [3,8,16]. Others argue that it is often not possible or appropriate to demonstrate benefit to the individual [17]. The Australian authorities allow emergency research if it is approved by the ethics committee as having an appropriate risk-benefit ratio, and if there is no obvious reason why the participant would refuse consent had they have been capable of consenting [18]. In New Zealand the Health Research Council require ethics committees to consider whether proposed waived consent is appropriate given the research circumstances [19,20].

### Approaching the Community

The FDA's substantive requirement for community consultation poses a significant challenge for emergency research [21]. Problems include varying definitions of what is meant by the term community for different studies[22,23], and lack of clarity in the aims and forms of consultation. It is recognized that community consultation is not about obtaining community consent or endorsement of research, nor does it serve as a proxy for consent or make an unethical study ethical. In some cases community consultation is confused with community information giving, or sensitisation. Although community consultation should provide investigators and institutional review boards with insight into aspects of the study, such as cultural concerns, that may not have been considered by the investigators, exactly what should be achieved with whom is a grey area. A workshop held in 2006 which evaluated the issues and concerns around the EFIC reached no consensus but called for more discussion and consideration of this question [24].

Community consultation and sensitisation activities might best focus on those most likely to be recruited into research, rather than general communities, many of whom will not be eligible or involved in a hospital based trial. A community consultation study by Morris and colleagues sought the views of members of several research communities (parents, healthcare workers) regarding a hypothetical randomised controlled trial of induced hypothermia in children [25]. Exception from full prior informed consent was found to be acceptable, but only if families were informed of the study prospectively and had the opportunity to decline participation of their child before enrolment.

### Vulnerable populations

Vulnerable subjects in research generally include children, prisoners, pregnant women, mentally disabled persons, or economically or educationally disadvantaged persons [26]. These groups are recognised to both require special protection from abuse and exploitation and to be included in research and associated benefits. In the setting of resuscitation research, all potential subjects are vulnerable by virtue of their lack of capacity to understand or communicate opinions regarding their participation in the research. In the case of a child who requires resuscitation, the accompanying parent or guardian acts as a surrogate. However, adequately informed permission, even by a surrogate decision-maker, is often impossible within the short therapeutic window required for a successful intervention (for example situations such as cardiac arrest, status epilepticus or life-threatening shock). Moreover, vulnerability in terms of difficulty in capacity to understand or communicate opinions can extend to surrogate decision makers in an emergency situation and undermines their ability to make an informed choice. In these situations the requirement for informed and voluntary parental permission may introduce a selection bias undermining the generalizability and feasibility of answering specific research objectives. In the case of a paediatric fluid resuscitation trial for the treatment of shock, the requirement for full prior IC from all surrogates would result in a trial conducted in a less critically ill cohort of children whose data may be less informative or even misleading for severely ill children.

### The FEAST Trial and Deferred Consent

From our review of regulatory situation for different regions and within the literature we found a lack of detailed guidance or previous examples to follow that were appropriate for this study. We were keen to ensure the following:

1. *There is written prior informed consent wherever possible.*Many, but importantly not all, of the children that are eligible for the FEAST trial are severely ill requiring emergency resuscitation and accompanied by parents or guardians who may be too distressed to give fully informed consent. If a child is stable enough to allow time and the parents or guardians are not too distressed to understand the information, ask questions and give or decline consent then the standard consent process applies.

2. *Incorporation of an option for deferred consent with prior assent*.

**Figure 1:**
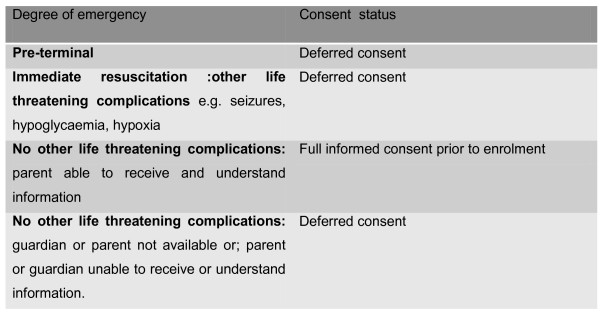
**Box 1 Criteria for deferred consent**.

For the trial to enrol, randomise and evaluate outcomes of the three different fluid resuscitation strategies it was important that the design included children across the severity range. We were concerned that limiting the trial to children who were not severely ill would result in potentially misleading findings for the target group (the most unwell children) as we would not obtained data from the children who are at the most at risk. Furthermore, excluding these children would then necessitate prohibitively large and expensive trials of potentially important therapeutic interventions as it would take longer for a definitive answer to be derived. We concluded that a process of deferred consent that incorporated verbal parental or guardian assent would be appropriate for a sub-group meeting set criteria (Figure 1). These criteria included surrogates who appeared to be too distressed to read and understand the information, ask questions and make an informed decision. More commonly in this resource-limited African setting many of surrogates are not literate and thus require the full information sheets read to them in the local language- which requires sufficient time. Based on past research and practice in the area [27,28] our perception was that a full consent process at enrolment was unlikely to result in a 'fully informed' decision among highly distressed surrogates; more likely to contribute to confusion and concern.

**Figure 2:**
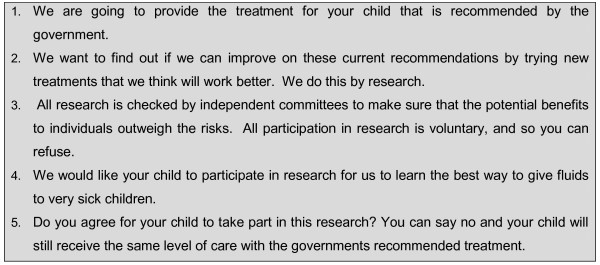
**Box 2 Phrasing of assent in the deferred consent process**.

Where deferred consent criteria were met, we were guided by previous research on informed consent in a rural African setting [27,28] and the findings of the Morris study [25] described above. The provision of basic awareness of the study in advance was one of our requirements so this gave all parents/surrogates, regardless of the condition of their child or the apparent levels of parental distress, an opportunity to decline participation of their child before enrolment. Parental or guardian 'assent' used in this context is distinct from that given by a minor for research, although it employs the same concept. The information given during this verbal assent process includes key elements of ICH-GCP (Figure 2). The surrogate decision makers of potentially eligible children are informed at admission that in addition to standard care, their child may be involved in research but not if they chose to opt out. Information is conveyed by a short verbal 'assent' process, and conducted at the point of enrolment by a specially trained study clinicians or nurses. Surrogates could request fuller study information at this stage if they wanted it. Verbal assent to the research, including randomisation, is followed by the full details of the study and request for formal written consent only when the child's medical condition has become less critical.

Deferred consent therefore encompasses all required details and relevant elements present in standard consent, the difference being that the process is split to give the minimum information verbally and followed by full details later once logistically or justly appropriate. Mostly importantly, the voluntary participation in research is respected.

3. *Early mortality - a challenge for deferred consent*. The *a priori *case fatality of the children eligible for FEAST is high (over 15%[29]) and a proportion of children enrolled following parental verbal assent die before full informed consent is obtained. We gave this predicted situation careful consideration. We did not want to cause more distress to these parents, yet these children's data were fundamental to the objectives of the trial, since early mortality is the primary endpoint. We opted not to approach parents for retrospective full, written informed consent in the event of death for three main reasons: a) parents would have been through an assent process and could request further trial information if they required it as part of inevitable discussion after a child's death; b) revisiting the full consent process might lead to parents blaming themselves for having given permission to participate in research when it would be difficult to explain retrospectively that fatal outcome was not unexpected consequence of severe illness; and c) the responsibility for ensuring overall patient benefit from such a trial and appropriate inclusion of children fell not with parents but with the trial teams and ethics review committees.

4. *Community consultation and sensitisation. *For this hospital based trial we discussed which communities to consult with and give information about the trial. Drawing on the literature presented in this paper, the focus of FEAST trial communication strategy was aimed at hospital management team and clinical and nursing staff, and to parents in the paediatric wards. Trial specific information was not circulated to chiefs and community leaders because there were concerns that this would potentially contribute to rumours and concerns, and more importantly because of the relatively small numbers of patients involved (Kenya) and the difficulty in identifying appropriate communities and representatives (Tanzania and Uganda). As part of the process of designing our community consultation and consent methods, the trial incorporated ideas from a workshop involving independent paediatricians and ethicists from the region (themselves representatives of 'a form of community').

5. *Formal and Standardised Staff Training*Essential to the successful implementation of the above communication and consent processes is that FEAST trial staff receive specific informed consent training. They are operationally assessed before and throughout the study. A concern is that staff may opt for verbal assent more than is strictly necessary, because this is easier to do at the point of enrolment than full prior written consent. We are therefore prospectively recording reasons for its use in the trial data. The process appears to be working well in practice, and is being regularly monitored by the trial management team. We are reassured that some parents are refusing trial enrolment either at point of verbal assent or consent. This suggests that, at least, some parents understand that they have a choice and are exercising this right. If parents subsequently decline written consent, which is usually when most of the interventions have been completed, children are withdrawn from the trial and the parents have the right to choose whether or not to allow the use of child's trial data to be used for the primary endpoint (48-hour survival).

## Discussion

Researchers and institutional review boards have questioned the interpretation and application of the 1996 Final Rule. They are concerned about whether research in the chaotic environment of the emergency room, or pre-hospital settings, provides participants with adequate protection. Others believe that current regulations are overprotective and restrictive, limiting important research [4]. If the patient would trust the physician to use unproven treatments as a routine therapy, then such trust could be presumed to extend to using the same treatment as part of a well designed and monitored study which had been independently peer-reviewed and endorsed by ethical approval.

By opting to use a process of deferred consent that incorporates obtaining verbal assent the FEAST trial is being conducted in accordance with the Nuffield Council on Bioethics guidelines, and other international guidelines [30,31]. It places more responsibility with the ethics committees to decide whether they were confident that the rights and well being of the participants are being protected. In East Africa there are no legal requirements to follow particular guidelines, but our institution works to ICH-GCP for all of our trials http://www.kemri-wellcome.org. Whilst there are local trial regulations to follow in each of the countries we are working in, none gave specific details about emergency research. The FEAST protocol, including the consent processes described above, has obtained full ethical approval in the UK and by three African committees.

## Conclusions

We consider it of value to share how we approached consent for this specific trial since severely ill children are under-represented in research and current regulations are challenging for researchers. The FEAST trial, to our knowledge, is the first and the largest late phase trial to have provided specific emergency consent process since the change in regulatory requirements. However, we recognise that there now needs to be a formal study to explore the views and experiences of different groups involved, including parents, research staff, trial monitors and managers, and members of ethics committees and regulatory agencies. In particular, we will be interested in documenting these key stakeholders' perceptions of the acceptability and appropriateness of deferred consent, and their recommendations for similar studies in future.

## List of Abbreviations

CIOMS: Council for International Organizations of Medical Sciences; EFIC: Exemption from informed consent; FDA: United States Food and Drug Administration; FEAST: Fluid Expansion As Supportive Therapy; IC: Informed consent; ICH-GCP: International Committee on Harmonisation Good Clinical Practice; UNESCO *United Nations Educational, Scientific and Cultural Organization*; US: United States of America.

## Competing interests

"The author has completed the Unified Competing Interest form at http://www.icmje.org/coi_disclosure.pdf (available on request from the corresponding author) and declare that (1) (KM, SM, MB, SK, TL) have no relationships with any companies that might have an interest in the submitted work in the previous 3 years; (2) ours spouses, or children have no financial relationships that may be relevant to the submitted work; and (3) (KM, SM, MB, SK, TL) have no non-financial interests that may be relevant to the submitted work."

## Authors' contributions

KM, SM and TL were responsible for the literature review and drafting the manuscript

KM MB, SM and TL were responsible for development of the approved consent process for the FEAST trial. All authors contributed to the writing of the manuscript and approval of the final draft
